# Chronic Expanding Hematoma in the Popliteal Fossa after Pseudoaneurysm Surgery because of Nail Puncture

**DOI:** 10.1155/2014/961691

**Published:** 2014-12-25

**Authors:** Serdar Yilmaz, Deniz Cankaya, Alper Deveci, Bulent Ozkurt, Mehmet Emin Simsek, Abdullah Yalcin Tabak, Murat Bozkurt

**Affiliations:** ^1^Department of Orthopaedics and Traumatology, Ankara Numune Training and Research Hospital, Ankara, Turkey; ^2^Department of Orthopaedics and Traumatology, Ankara Ataturk Training and Research Hospital, Ankara, Turkey

## Abstract

Hematomas caused by surgery or trauma that persist and expand slowly for more than a month are defined as chronic expanding hematomas (CEH). Magnetic resonance imaging (MRI) is useful for the diagnosis. Total excision with the pseudocapsule is the treatment method. Pseudoaneurysms result from arterial wall disruptions and can be mistaken for CEH. We present a rare case report of a 45-year-old man with a large, painful swelling in his left popliteal fossa. He had a puncture wound by a nail 11 years ago and a gradually expanding mass occurred in his popliteal fossa. A pseudoaneurysm was detected and operated a year later. After surgery, a gradually expanding mass recurred in his popliteal fossa. On the arteriography, the popliteal artery was occluded and the blood flow was maintained with collateral vessels. On MRI, an enormous swelling of 115 × 107 × 196 cm in diameter was seen. It was diagnosed as CEH and was excised completely protecting the collateral vessels and there was no recurrence after a year from the surgery.

## 1. Introduction

Hematomas caused by surgery or trauma usually resolve without sequelae. However, these formations occasionally may persist and expand slowly in more than a month. Such kind of hematomas is defined as “chronic expanding hematomas” (CEH) [1]. Trauma against the wall of the artery may lead to the development of a pseudoaneurysm and it must be kept in mind in the differential diagnosis of CEH. We present a complicated case which has been operated because of popliteal swelling after nail puncture which was diagnosed as pseudoaneurysm and later operated for CEH.

## 2. Case Presentation

**Figure 1:**
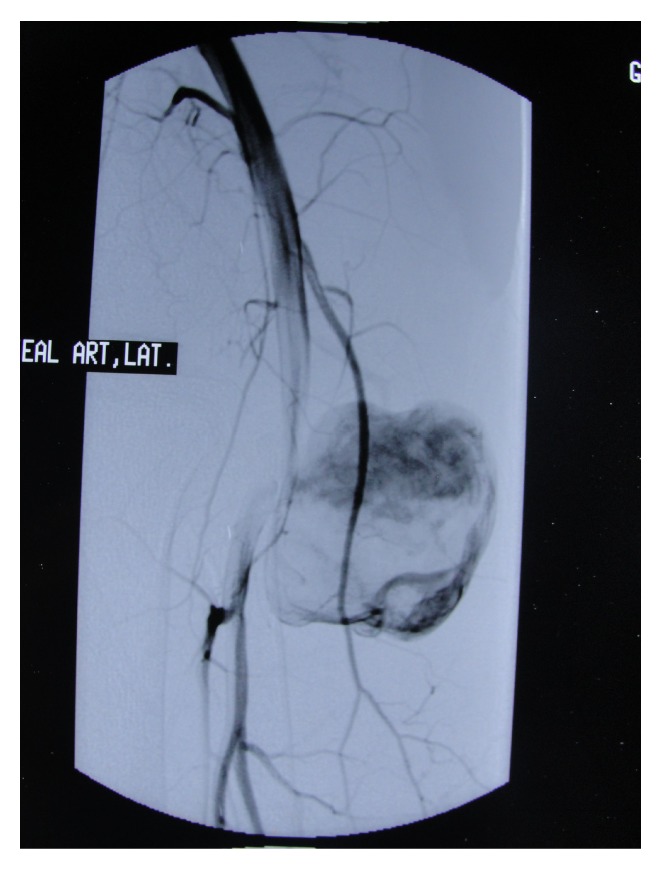
The arteriography of the popliteal artery showed the pseudoaneurysm.

A 45-year-old man was presented in our department with a large, painful swelling in the popliteal fossa in his left leg. There has been a puncture wound by a nail 11 years ago and he resorted to another hospital. At that institution, medical treatment was given in accordance with the patient's momentary condition. Nevertheless, a slowly growing mass occurred in his popliteal fossa in a year following being subjected to the puncture wound. He resorted to cardiovascular surgery department in another hospital and Doppler ultrasonography together with arteriography was taken. A popliteal pseudoaneurysm was detected in the distal part of the popliteal artery near the bifurcation to the anterior and posterior tibial arteries and was about 4 cm in diameter (Figure 1). The patient was taken into operation by cardiovascular surgeons after the angiography. He had an excision of the pseudoaneurysm and primary repair surgery to popliteal artery. After surgery, the popliteal artery was patent according to Doppler USG. He had a foot drop after the operation which resolves spontaneously after a year. At early follow-up, a gradually expanding mass recurred in the popliteal fossa. He went to cardiovascular surgery with this complain, but observation was recommended. Monophasic flow was seen on the popliteal artery according to Doppler ultrasonography which was taken 5 years after the operation. He resorted to some medical centers for his swelling to be treated during 10 years after bypass surgery, but observation without any interference was suggested according to the patient's momentary condition. He applied to our department with a huge popliteal mass associated with obstructed popliteal artery.

**Figure 2:**
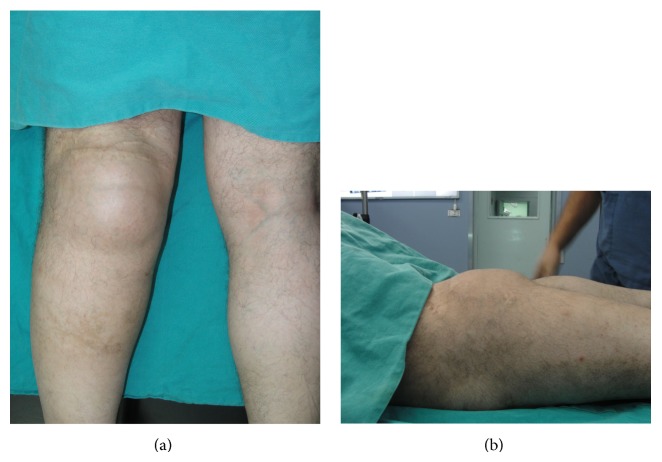
The posterior and lateral view of the popliteal mass on the operating table.

**Figure 3:**
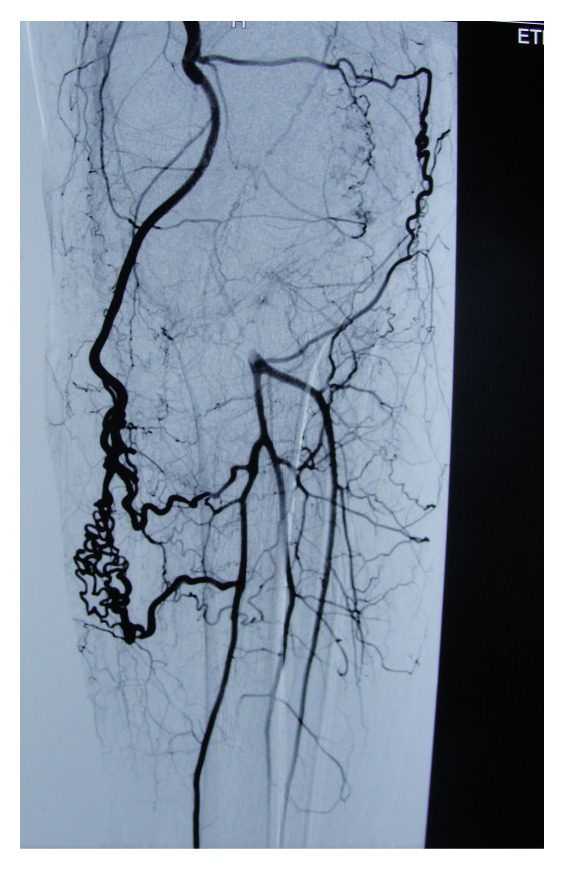
The arteriography of the popliteal mass which showed the occluded popliteal artery and the distal flow with collateral vessels.

**Figure 4:**
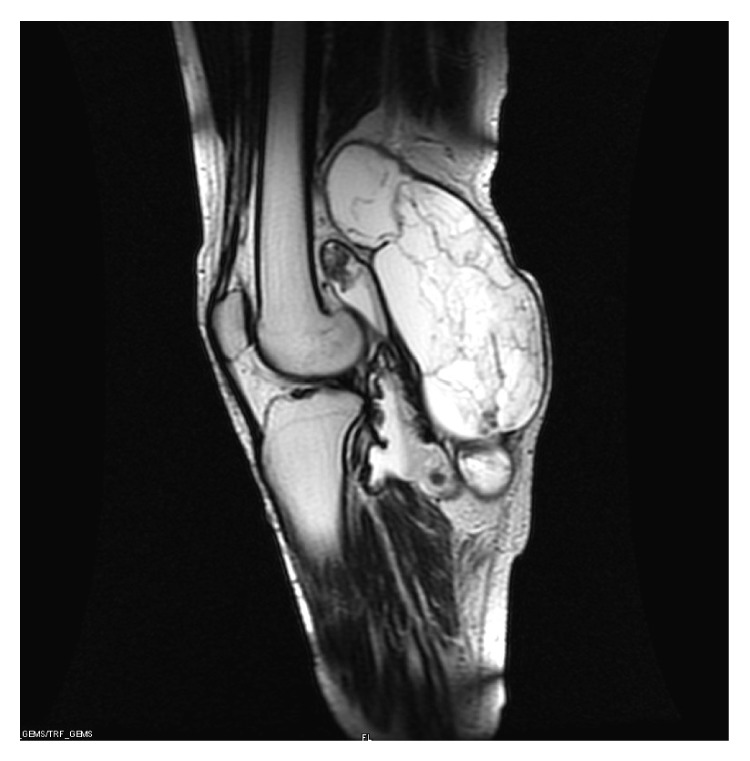
The sagittal section of the popliteal mass on MRI showed the enormous mass.

On the physical examination of the patient, there was a large mass (28 × 10 cm) in the popliteal fossa with old surgical scars. The lesion was soft, fluctuant, and painless in palpation (Figure 2). The neurological examination of the lower leg was normal. The blood tests were normal. In the arteriography images, the popliteal artery occluded in the proximal section but was filled in the distal section with collateral vessels (Figure 3). The MRI scan revealed an enormous soft tissue mass of 115 × 107 × 196 cm in size between the medial and lateral heads of the gastrocnemius with the features compatible with hematoma with lobulation (Figure 4).

**Figure 5:**
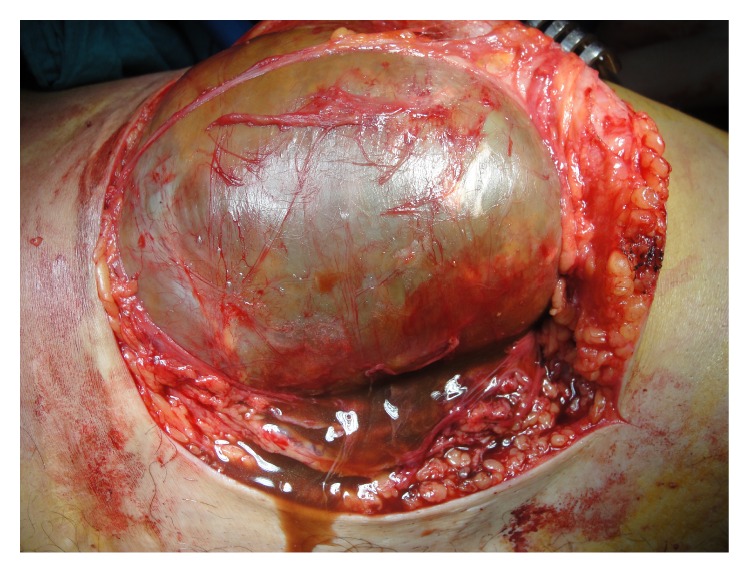
Intraoperative photograph of the mass and chocolate-brown fluid was seen.

**Figure 6:**
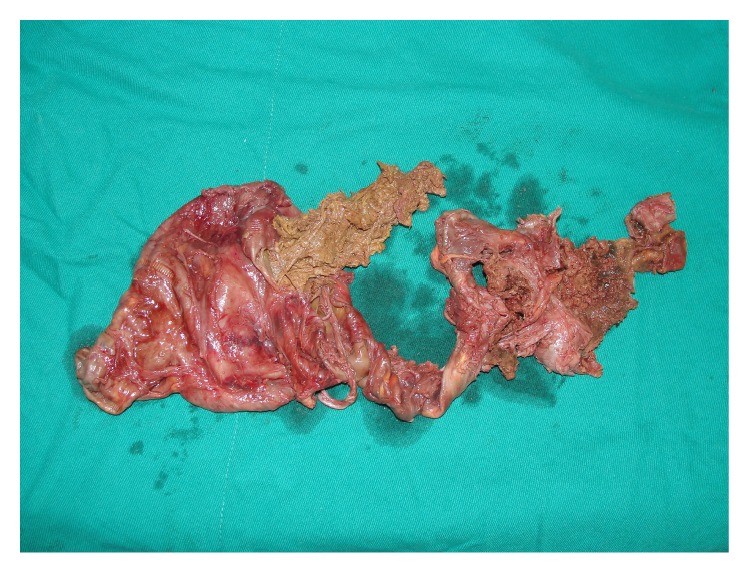
The excised CEH with villous formation.

**Figure 7:**
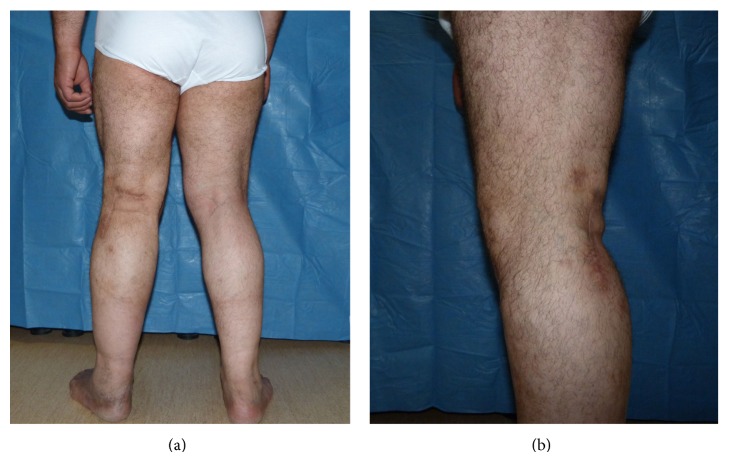
Lateral and posterior view of the patient one year after the surgery.

The mass is excised from the adhered tibia protecting the nerve and the collateral vessels with hockey stick incision on the popliteal fossa on prone position (Figure 5). There was about 1,5 L of chocolate-brown fluid evacuated from a well-defined wall and a multilocular cyst-like appearance was seen. The findings we gathered from the operation were compatible with a large fibrous cavity with villous formation containing a considerable quantity of altered blood clot (Figure 6). A complete resection of the pseudocapsule was performed. The underlying fascia was extendedly sutured with the subcutaneous tissue, in order to avoid any dead space where new hematoma can develop. The liquid and soft tissue culture was sterile. The histopathological examination revealed abundant fibrous tissue with the features of hemorrhage. After one year from the surgical treatment, the patient has not shown any sign of recurrence (Figure 7).

## 3. Discussion

Most hematomas subside without causing any serious clinical problem. However, in some cases, the hematomas may persist for longer periods and come out to be slowly expanding lesions in soft tissues simulating neoplastic growth [2]. Such kind of hematoma that persists and increases in size more than a month after the initial hemorrhagic event is defined as “chronic expanding hematoma” by Reis et al. [1]. If the hematoma is large it results in displacement of skin and subcutaneous fatty tissue from more deeply located fixed fascia creating a potential space with formation of blood filled cysts and may not be completely resorbed [3]. Then, it is encapsulated by a fibrous wall forming a chronic swelling. The vasoactive substances and the breakdown products in the blood induce an inflammatory reaction and this reaction activates capillary permeability and causes additional bleeding from fragile newly formed capillaries in the granulation tissue and the hematoma expansion occurs [2–4].

Magnetic resonance imaging and the histopathological examination are crucial for the diagnosis [3]. The correct treatment for the CEH is surgical excision, including the pseudocapsule. Aspiration of the liquid, drainage, and curettage could result in serious bleeding from the hypervascular subcapsular lesion and have a higher possibility of recurrence or a chronic draining sinus with or without infection [5, 6]. On the other hand, complete resection of the pseudocapsule and a meticulous suture of the subcutaneous tissue, with the underlying fascia in order to eliminate the dead space, are highly recommended [7].

The region around the knee is the most common presenting site for the development of a pseudoaneurysm. Pseudoaneurysms are usually progressive and have complications, including thrombosis, embolization, and rupture, and should be treated by surgery [8]. The operated artery can be occluded rarely after surgery at follow-up as in our case. After appropriate surgical method was applied, hemostasis becomes very significant as, in our case, we came up against a slowly expanding mass which recurred in his popliteal fossa.

It is essential to distinguish the pseudoaneurysms from the CEH when a mass was detected. On physical examination, if the mass bears the characteristics of a pulsatile mass and gives thrills, the possibility of the presence of pseudoaneurysm or arteriovenous fistula should be taken into consideration. The differentiation may not be possible macroscopically all the time; at this point an arteriography must be demanded to differentiate between pseudoaneurysm and CEH. The differential diagnosis of the CEHs includes popliteal artery aneurysms, arteriovenous malformations, hemorrhagic soft tissue tumor (hemangiopericytoma, cavernous hemangioma), sarcoma, synovial chondromatosis, actinomycosis, and inflammatory pseudotumor [1, 7, 9].

In the literature, only one case associated with these two diseases has been reported. It was because of applying biopsy to the pseudoaneurysm on the axilla [10].

We conclude that CEH must be kept in mind when slowly expanding popliteal swellings after surgery was seen. MRI and arteriography are the two major diagnostic methods to exclude the associated potential vascular problems. A careful preoperative plan and a meticulous surgical treatment on CEH could limit the possibility of recurrence considerably.
